# Philosophical Transactions of the Royal Society of London, Vol. LXXI. For the Year 1781. Part II

**Published:** 1782

**Authors:** 


### LONDON MEDICAL JOURNAL,

For APRIL, MAY, and JUNE 1782.

### SECTION I.

### BOOKS.

I. *Philoſophical Tranſactions of the Royal Society of London, Vol.* LXXI. *for the Year* 1781. *Part* II. 4*to. London,* 1782, 318 pages, with 17 copper plates.

OF the firſt part of this volume we gave an account in our Journal for September 1781. This ſecond part contains twenty papers, but of theſe we ſhall content ourſelves with giving abſtracts of the three following:

I. *Some calculations of the number of accidents or deaths which happen in conſequence of parturition; and of the proportion of male to female children, as well as of twins, monſtrous productions, and children that are dead born; taken from the*

*Midwifery reports of the Weſtminſter General Diſpenſary: with an attempt to aſcertain the chance of life at different periods, from infancy to twenty-ſix years of age; and likewiſe the proportion of natives to the reſt of the inhabitants of London. In a letter from* Robert Bland, M. D. *phyſician man-midwife to the Weſtminſter General Diſpenſary, to* Samuel Foart Simmons, M. D. F. R. S.

Dr. Smellie has curſorily mentioned, for the encouragement of his pupils, the ſmall proportion of præternatural and laborious births to the natural; but he omitted to extend his views farther, or to point out the proportionate number of conſequent accidents, which might occur to retard or prevent the recovery of the woman, although this is not leſs neceſſary to be known than the former. With a view to theſe and other uſeful purpoſes, we are here preſented with a variety of intereſting *data* founded on a regiſter kept by the author at the Weſtminſter General Diſpenſary, from its firſt inſtitution in 1774 to 1781, and in which he has carefully noted, 1. The ages of the ſeveral women; 2. the number of children they had borne; 3. the ſexes of the children; 4. the number of children they had been able to preſerve; 5. the place or country where they and their huſbands were born; 6. the

6. the accidents that attended, or were the consequences of parturition; 7. the fexes of the children delivered; 8. the number of twins or triplets; 9. the number of children that were deficient or monftrous; 10. the number of children that were dead born, or (where the account could be procured with certainty) who died within four or five weeks from their birth.

From this regifter Dr. Bland has compofed feveral interefting tables, which, together with his comments on each, are well deferving the public attention.

The firft table is calculated to fhew the proportion of difficult labours, and of the accidents or deaths that happen in confequence of parturition. From this table it appears that of 1897 women, 1792 had natural labours, not attended with any particular accident; of the remaining 105, 63, or 1 in 30, had unnatural labours; in 18 of thefe, or 1 in 105, the feet prefented; in 36, or 1 in 52, the breech; in 8 the arms; and in 1 the funis prefented. Seventeen women, or 1 in 111, had laborious labours: in 8 of thefe, or 1 in 236, the heads of the children were leffened; in 4 a fingle blade of the forceps was ufed; and in the remaining 5, in which the faces of the children were turned to the pubes, the delivery was accompliſhed by the pains. One woman had convulſions about the ſeventh month of her pregnancy, and was delivered a month after of a dead child, and recovered. Another had convulſions during labour; brought forth a live child, and recovered. Nine women, or 1 in 210, had uterine hemorrhage before and during labour; of theſe, one died undelivered; another a few hours, and a third ten days after delivery, but the other ſix recovered. Five women had the puerperal fever, of whom four died. Two were ſeized with mania, but recovered in about three months. In one woman a ſuppuration took place, ſoon after labour, from the vagina into the bladder and rectum; this patient recovered, but the urine and ſtools continued to paſs through the wounds. In another woman the perinæum was lacerated to the ſphincter ani. A ſuture was attempted, but without effect; ſhe recovered, but is troubled with prolapſus uteri. Five others had large and painful ſwellings of the legs and thighs but recovered.

In two women, we are told, the uterus was retroverted in the third or fourth month of pregnancy; but in both was replaced without any bad conſequence.

Beſides the accidents above-mentioned, Dr.

Bland has thought it right to inform us, that many of the patients were afflicted with fevere after-pains, or had what is called the milk fever. Neither of thefe ever proved fatal, and upon the whole he is difpofed to think, that the lower fort of people recover more certainly after parturition than perfons in higher ftations of life; at leaft, he obferves, they are lefs fubject to the puerperal fever, which is fo fatal, if not checked on its firft attack; and which, if not caufed, is certainly nourifhed, and its malignancy increafed by great fires, clofe rooms, warm feptic diet and coftivenefs.

The fecond table contains the proportion of male to female children, the number of twins, and of the children that were deficient, monftrous, or dead born. It feems that 1897 women were delivered of 1923 children; 972 boys, and 951 girls. Twenty-three of the women, or 1 in 80, were delivered of twins, 16 of whom were boys, and 30 girls. One woman was delivered of 3 girls. Eight of the children, or 1 in 241, were deficient or monftrous. Of thefe one was web fingered, another had a hair lip, a third had a dropfical head and diftorted fpine, and a fourth fimply a dropfical head. In one a part of the palate, and in two others a confiderable rable portion of the cranium was wanting. The eighth had two heads. Dr. Bland's account of this extraordinary fœtus is accompanied by a very elegant engraving by Bafire, as is likewife his defcription of another fingular monftrous production. Eighty-four of the children, or 1 in 23 of the whole number were dead born *, of thefe 49 were boys and 35 were girls.

Of 1400 women who returned their letters, or of whom a certain account could be obtained, 85, or nearly 1 in 16, had buried their children before the end of two months. Of the number dead in this period 53, or 5 in 8, were boys, and 32 girls.

This fingular circumftance of there being a greater number of males than females among the ftill-born children, and of a greater number of male children dying in infancy than of females, has been remarked by Dr. Price and other writers on calculations; and Dr. Haygarth has fhewn that at Chefter more hufbands die in a given period than wives. This natu-

* By dead-born children, Dr. Bland means thofe which die after they have been perceived to move, that is generally after five months. Abortions or deaths before that period, he thinks, may reafonably be eftimated at double this number; fo that, perhaps, 1 child in 8 dies in the womb, or in the act of coming into the world.

rally rally fuggefts an enquiry, whether the lives of males are at all ages more precarious than thofe of females? To be enabled to affift in anfwering this queftion, our author has added the following article to his regifter, viz. of the number of children that fhall be living at the time the women apply for their letters, how many will be boys and how many girls? The refult of this enquiry will probably be the fubject of a future paper.

The third table relates to the ages at which women begin and ceafe to be capable of bearing children, and to the intermediate periods at which they are moft fo.—It appears that of 2102 pregnant women, 85* were from 15 to 20 years of age; 578 from 21 to 25; 699 from 26 to 30; 407 from 31 to 35; 291 from 36 to 40; 36 from 41 to 45; and 6 from 46 to 49.

We are next prefented with two tables begun fome time after the preceding ones, and which fhew the number of children borne by 1389 women, with the number that were living at the

* Thirty-fix of thefe, the author obferves, were from 15 to 19, and of thefe one was between 15 and 16; a fecond between 16 and 17; three between 17 and 18; ten between 18 and 19; and twenty-one between 19 and 20.

time of their applying to the Difpenfary. Thefe tables ferve to prove how exceedingly fertile the women of the poorer claffes in this country are, and at the fame time how unable to rear any confiderable number of children; for although 321 of the women had borne fix children and upwards each, and were all again pregnant, 19 only of them had been able to rear fix or more children; and although 102 of the women had borne nine children and upwards each, only one of them had been able to preferve that number living. Our author attributes this great mortality amongft the children to the poverty of the parents, which prevents their taking the neceffary care of, or even affording fufficient nourifhment and cloathing to their offspring.

We are next prefented with fome ingenious fpeculations relative to the chance of life from infancy to 26 years of age in London. From our author's inquiries on this fubject he fuppofes that of 5400 perfons only 1620, or 3-10ths would be living at the end of 26 years.

The paper clofes with a comparative table of the population of London, from which it appears that of 3236 married perfons, 824 were born in London; 1870 in the different counties of

Eng-

England and Wales; 209 in Scotland; 280 in Ireland; and that 53 were foreigners.

Dr. Bland obſerves, that of thoſe born in London, there were one-fifth more women than men. This he accounts for by ſuppoſing that a greater number of males die or migrate before they attain a marriageable age than women. He alſo finds that of the Scotch and of the foreigners the women are in proportion to the men as about 1 to 3; but that of the Iriſh they are as 3 to 7.

II. *Account of a child who had the ſmall pox in the womb. In a letter from* William Wright, M. D. F. R. S. *to* John Hunter, Eſq. F. R. S.

From the obſervations adduced by Mr. Hunter in the *Philoſophical Tranſactions*, vol. 70, and by Dr. Bland in the *London Medical Journal*, vol. 2. it ſeems clearly to be proved that the foetus is capable of receiving the variolous infection *in utero*. This curious fact is ſtill further corroborated by the paper before us. It relates to a female negro in Jamaica, who had the ſmall pox in the natural way when big with child. They were few, diſtinct and large, and ſhe went through the diſeaſe with very little trouble, till on the fourteenth day from the eruption ſhe was attacked with a fever, which laſted only a few

 hours.

hours. She was, however, the ſame day taken in labour, and delivered of a female child with the ſmall pox on her whole body, head, and extremities. They were diſtinct and very large, ſuch as they commonly appear on the eighth or ninth day in favourable caſes. The infant died the third day after its birth, but the mother recovered.

Dr. Wright adds, that in the courſe of many years practice in Jamaica, he has remarked, that where pregnant women have been ſeized with the natural ſmall pox, or been by miſtake inoculated, they have generally miſcarried in the time of, or ſoon after, the eruptive fever; but that he never ſaw any ſigns of ſmall pox on the bodies of any of the children, excepting the one above mentioned.

III. *Experiments on the power that animals, when placed in certain circumſtances, poſſeſs of producing cold. By* Adair Crawford, M. D. *Communicated by Sir* Joſeph Banks, *Bart. P. R. S.*

From the firſt dawnings of philoſophy it muſt have been perceived, that moſt animals have a higher temperature than the medium in which they live; and that a conſtant ſucceſſion of freſh air is neceſſary to the ſupport of animal life. The cauſes of theſe phenomena have afforded matter matter for much ſpeculation in ancient as well as modern times: but the diſcovery that animals have, in certain circumſtances, the power of keeping themſelves at a lower temperature than the ſurrounding medium was reſerved for the induſtry of the preſent age.

This diſcovery ſeems originally to have ariſen from obſervations on the heat of the human body in warm climates. It was mentioned by Governor Ellis in 1758; it was taught by Dr. Cullen before the year 1765; and at length it was completely eſtabliſhed by the experiments of Dr. Fordyce in heated rooms, which were laid before the Royal Society in 1774.

Various opinions have been entertained with regard to the cauſe of the facts which were eſtabliſhed by theſe experiments; ſome have attributed the cold ſolely to evaporation; others have maintained that it did not ariſe ſolely from this cauſe, but depended partly upon the energy of the vital principle, being greater than what would have been produced by an equal maſs of inanimate matter.—The experiments before us were made with a view to determine this point with greater certainty. The ingenious author has prefaced his account of them with a few remarks on the progreſſive improvements which have been made in the knowledge of heat in general.

To difcover whether the cold produced by a living animal placed in air hotter than its body, be not greater than what could be produced by an equal mafs of inanimate matter, Dr. Crawford took a living frog, equally moift, and of nearly the fame bulk, the former of which was at 67°, and the latter at 68°, and laid them upon flannel in air which had been raifed to 160°; in two minutes, when the air was at 102°, the dead frog was at 72°, and the living frog at 68°, and at the end of 25 minutes the dead frog was at 81¼ and the living frog at 78¼, the temperature of the air being then at 95°. The internal heat of both animals was found to be the fame with that at the furface.—In this experiment the living frog acquired heat more flowly than the dead one. Hence our author concludes that its vital powers muft have been active in the generation of cold.

To determine whether the cold produced in this inftance depended folely upon the evaporation from the furface, increafed by the energy of the vital principal, a living and dead frog were taken at 75°, and immerfed in water at 93°, the living frog being placed in fuch a fituation ation as not to interrupt refpiration.—In one minute the dead frog was at 85° and the living one at 81°; at the end of 5, 6, and 8 minutes the heat of both was ftationary, that of the dead frog being 91½°, and that of the living frog 89°.—In a note to this experiment the author obferves, that the water, by the cold frogs and the agitation it fuffered during their immerfion, was reduced nearly to 91½°.

Thefe experiments prove that living frogs have the faculty of refifting heat, or producing cold, when immerfed in warm water, while the experiments of Dr. Fordyce prove, that the human body has the fame power in a moift as well as in a dry air; Dr. Crawford therefore thinks it highly probable that this power does not depend folely upon evaporation.

He has obferved that healthy frogs, in an atmofphere above 70°, keep themfelves at a lower temperature than the external air, but are warmer internally than at the furface of their bodies; for when the air was 77°, a frog was found to be 68°, the thermometer being placed in contact with the fkin; but when the thermometer was introduced into the ftomach, it rofe to 70½°.

He has likewife found that an animal of the fame fame fpecies placed in water at 61°, was nearly 61¼° at the furface, while internally it was 66½°. Thefe obfervations, however, we are told, extend only to frogs living in air or water at the common temperature of the atmofphere in fummer, as they do not hold good with refpect to thofe animals, when plunged fuddenly into a warm medium, as in the preceding experiments.

To determine whether other animals alfo have the power of producing cold, when furrounded with water above the ftandard of their natural heat, a dog at 102°, was immerfed in water at 114°, the thermometer being clofely applied to the fkin under the axilla, and fo much of his head being uncovered as to allow him a free refpiration.

In 5 minutes the dog was 108°, water 112°.

| | | | |
|---|---|---|---|
| 6 | —— | 109 | 112 |
| 11 | —— | 108 | 112 |

the refpiration having become very rapid.

In about half an hour the dog was 109°, water 112°, the animal was then in a very languid ftate.

Small quantities of blood being drawn from an artery and a vein, the temperature did not feem fo much increafed above the natural ftandard, and the fenfible heat of both feemed to be nearly the fame.

ſame. The venous blood, however, inſtead of its uſual dark red hue, had acquired very nearly the bright florid colour of the arterial; the animal which was the ſubject of this experiment had been previouſly weakened by loſing a conſiderable quantity of blood a few days before. When the experiment was repeated with dogs which had not ſuffered a ſimilar evacuation, the change in the colour of the venous blood was more gradual, but was ſtill ſo remarkable, that the blood which was taken in the warm bath could readily be diſtinguiſhed from that which had been taken from the ſame vein before immerſion by thoſe who were unacquainted with the motives or circumſtances of the experiment.

To diſcover whether a ſimilar change would be produced in the colour of the venous blood, in hot air, a dog at 102° was placed in air at 134°.

In ten minutes the temperature of the dog was 104½°, that of the air being 130°. In fifteen minutes the dog was 106°, the air 130°; a ſmall quantity of blood was then taken from the jugular vein, the colour of which was ſenſibly altered, being much lighter than in the natural ſtate.—Dr. Crawford is inclined to conſider this effect of external heat upon the colour of venous blood, as a confirmation of the following opinion, which was firſt ſuggeſted to him by Mr. Wilſon of Glaſgow, viz. admitting that the ſenſible heat of animals depends upon the ſeparation of abſolute heat from the blood by means of its union with the phlogiſtic principle in the minute veſſels, may there not be a certain temperature at which that fluid is no longer capable of combining with phlogiſton, and at which it muſt of courſe ceaſe to give off heat?

From the preceding facts our ingenious author proceeds to explain what appears to him to be the true cauſes of the cold produced by animals when placed in a temperature which is above the ſtandard of their natural heat.

He begins with recapitulating ſome paſſages of his Eſſay on animal heat publiſhed ſome time ago, and in which having attempted to prove that animal heat depends upon the ſeparation of elementary fire from the air in the proceſs of reſpiration, he obſerved, that when an animal is placed in a warm medium, if the evaporation from the lungs be increaſed to a certain degree, the whole of the heat ſeparated from the air will be abſorbed by the aqueous vapour.

From the experiments on venous and arterial blood, recited in the third ſection of that work, it appears, that the capacity of the blood for containing heat is fo much augmented in the lungs, that, if its temperature were not fupported by the heat which is feparated from the air, in the procefs of refpiration, it would fink 30°. Hence, we are told, if the evaporation from the lungs be fo much increafed as to carry off the whole of the heat that is detached from the air, the arterial blood when it returns by the pulmonary vein will have its fenfible heat greatly diminifhed, and will confequently abforb heat from the veffels that are in contact with it and from the parts adjacent. Dr. Crawford thinks, that the heat which is thus abforbed in the greater veffels, will again be extricated in the capillaries, where the blood receives a frefh addition of phlogifton. If in thefe circumftances, the blood during each revolution were to be equally impregnated with this latter principle, it is manifeft, that the whole effect of the above procefs would be to cool the fyftem at the center, and to heat it at the furface. But from our author's experiments which have been laft recited, it appears that in a heated medium the venous blood, inftead of acquiring the dark livid hue which Dr. Prieftley with good reafon fuppofes to depend upon its combination with phlogifton in the minute veffels, becomes gradually paler and paler in its colour. From this circumſtance Dr. Crawford thinks we may conclude, that in a heated medium the blood does not attract an equal quantity of the phlogiſtic principle, and that as the quantity of heat given off by the blood in the capillaries will not be equal to that which it had abſorbed in the greater veſſels, poſitive cold will be produced. Thus, if the blood, for example, in its paſſage to the capillaries, abſorb from the greater veſſels a quantity of heat as 30°, and in conſequence of its being leſs impregnated with phlogiſton than formerly, it gives off at the extreme veſſels a quantity of heat only as 20°, a degree of refrigeration will be produced as 10°, and this cauſe of refrigeration will continue to act while the venous blood is gradually aſſuming the hue of the arterial, till the difference between them is obliterated, after which it will ceaſe to operate. Hence it appears, that when animals are placed in a warm medium, the ſame proceſs which formerly ſupplied them with heat, becomes for a time the inſtrument of producing cold.

Upon the whole, our author conſiders the increaſed evaporation, the diminution of that power by which the blood in the natural ſtate is impregnated with phlogiſton, and the conſtant reflux flux of the heated fluids towards the internal parts, as the great caufes upon which the refrigeration depends.

Having found that the attraction of the blood to phlogifton was diminifhed by heat, he thought it probable, on the other hand, that it would be increafed by cold. To determine this, a dog at 100° was immerfed in water nearly at 45°. In about a quarter of an hour a fmall quantity of blood was taken from the jugular vein, which was evidently much deeper in its colour than that which had been taken in the warm bath.

Dr. Crawford obferves, that from this experiment, compared with thofe which have been recited above, we may perceive the reafon why animals preferve an equal temperature, notwithftanding the great variations in the heat of the atmofphere; for as foon as by expofure to external cold, an unufual diffipation of the vital heat is produced, the blood begins to be more deeply impregnated with the phlogiftic principle: that it will therefore furnifh a more copious fupply of this principle to the air in the lungs, and will imbibe a greater quantity of fire in return: that in fummer on the contrary, the reverfe of this will take place, and hence the power of generating heat is in all cafes proportioned to the demand; and laftly, that from the changes which are produced in the colour of the venous blood by heat and cold, we may likewife perceive the reafon why the temperature of the body is frequently increafed by plunging fuddenly into cold water, and why the warm bath has fuch powerful effects in cooling the fyftem, and in removing a general or partial tendency to inflammation.

The other papers contained in this volume are, *Experiments upon gunpowder, by* Benjamin Thompfon, *Efq.* F. R. S. —*An account of a luminous appearance in the heavens, by Mr.* Tib. Cavallo, F. R. S.—*Of an earthquake at Hafodunos near Denbigh, by* John Lloyd, *Efq.* F. R. S. —*On the heat of the water in the Gulf ftream, by* Charles Blagden, M. D. F. R. S.—*Of the appearance of the foil on opening a well at Hanby in Lincolnfhire, by Sir* Henry C. Englefield, *Bart.* F. R. *and* A. S.—*Aftron. obfervations, by* Nath. Pigott, *Efq.* F.R.S.—*Abftract of a regifter of the barometer, thermometer, and rain, at Lyndon in Rutland,* 1780, *by* Thomas Barker, *Efq.*—*Natural hiftory of the infect which produces the gum lacca, by Mr.* James Kerr *of Patna.* —*Account of a phenomenon obferved upon the ifland of Sumatra, by* William Marfden, *Efq.*—*Farther experiments on cold, made at the Macfarlane obfer-*

*vatory*

*vatory belonging to Glasgow college, by* Patrick Wilson, M. A. (see Vol. I. of our Journal, page 443)—*A general theory for the mensuration of the angle subtended by two objects, &c. by* George Atwood, M. A. F. R. S.—*An account of the Ophidium barbatum* Linnæi, *by* P. M. Augustus Broussonet, M. D.—*A further account of the usefulness of washing the stems of trees, by Mr.* Robert Marsham, *of Stratton,* F. R. S.—*Hints relating to the use which may be made of the tables of natural and logarithmic sines, tangents, &c. in the numerical resolution of adfected equations, by* William Wales, F. R. S.—*Account of a comet, by Mr.* Herschel, F. R. S.—*A letter from Mr.* Joseph Willard, *concerning the longitude of Cambridge in New England.*—*An account of some thermometrical experiments, by* Tiberius Cavallo, F. R. S.

